# Multi-Responsive Optimization of Novel pH-Sensitive Hydrogel Beads Based on Basil Seed Mucilage, Alginate, and Magnetic Particles

**DOI:** 10.3390/gels8050274

**Published:** 2022-04-27

**Authors:** Natwat Srikhao, Korrapat Chirochrapas, Nessaraporn Kwansanei, Pornnapa Kasemsiri, Artjima Ounkaew, Manunya Okhawilai, Chutiwat Likitaporn, Somnuk Theerakulpisut, Hiroshi Uyama

**Affiliations:** 1 Department of Chemical Engineering, Faculty of Engineering, Khon Kaen University, Khon Kaen 40002, Thailand; natwat_s@kkumail.com (N.S.); korrapatchi@gmail.com (K.C.); nessaraporn.k@kkumail.com (N.K.); artjima.o@kkumail.com (A.O.); 2 Center of Excellence in Responsive Wearable Materials, Chulalongkorn University, Bangkok 10330, Thailand; 3 Metallurgy and Materials Science Research Institute, Chulalongkorn University, Bangkok 10330, Thailand; 4 International Graduate Program of Nanoscience & Technology, Chulalongkorn University, Bangkok 10330, Thailand; 6388315520@student.chula.ac.th; 5 Energy Management and Conservation Office, Faculty of Engineering, Khon Kaen University, Khon Kaen 40000, Thailand; somthe@kku.ac.th; 6 Department of Applied Chemistry, Graduate School of Engineering, Osaka University, Suita 565-0871, Japan; uyama@chem.eng.osaka-u.ac.jp

**Keywords:** hydrogel beads, Taguchi’s method, Grey relational analysis, drug delivery system

## Abstract

Conventional drug delivery systems often cause side effects and gastric degradation. Novel drug delivery systems must be developed to decrease side effects and increase the efficacy of drug delivery. This research aimed to fabricate hydrogel beads for use as a drug delivery system based on basil seed mucilage (BSM), sodium alginate (SA), and magnetic particles (MPs). The Taguchi method and Grey relational analysis were used for the design and optimization of the hydrogel beads. Three factors, including BSM, SA, and MPs at four levels were designed by L-16 orthogonal arrays. BSM was the main factor influencing bead swelling, drug release rate at pH 7.4, and release of antioxidants at pH 1.2 and 7.4. In addition, SA and MPs mainly affected drug loading and drug release rate in acidic medium, respectively. Grey relational analysis indicated that the composition providing optimal overall properties was 0.2 vol% BSM, 0.8 vol% SA, and 2.25 vol% MPs. Based on the findings of this work, BSM/SA/MPs hydrogel beads have the potential to be used as a pH-sensitive alternative material for drug delivery in colon-specific systems.

## 1. Introduction

Engineered materials that can control drug release have received a great deal of attention from researchers. These materials overcome disadvantages in drug delivery due to deterioration in the digestion system or to drug residues in the stomach affecting liver function. These problems cause the organs to work harder to remove residues; therefore, there is a need for new materials to control and optimize drug release in both dosage and time. This could reduce side effects of medication [1] such as damage to non-target cell tissues during delivery and excessive drug intake causing residues in the body [2].

Hydrogels are 3D structured materials that can easily retain and deliver drugs due to their high absorption ability and good compatibility with human tissues [1,3,4,5]. Hydrogels can be natural or synthetic polymers in a variety of forms, such as films [6], nanoparticles [7], microparticles [8], and beads [9]. Mucilage is a natural products of plant metabolism with low toxicity and high viscosity [1]. Consequently, mucilages can be suitable for use in gel-forming and as stabilizers in the food and pharmaceutical industries. Basil (*Ocimum basilicum* L.) is a plant native to Iran that is commonly grown in Thailand. Basil seeds are used in traditional medicine to treat inflammation, diarrhea, indigestion, and other diseases [2]. In addition, basil slime has been used in pharmaceuticals via preparation as a capsule laxative [10]. When it comes into contact with water, the outer layer of a basil seed quickly swells and forms a gelatinous substance, basil seed mucilage (BSM). BSM has two main components, i.e., 43% glucomannan and 24% (1,4)-linked xylan, along with 6% uronic acid, which forms hydrogen bonds with water molecules. These components provide a large amount of water storage. When water is retained, mucilage forms a chain of polysaccharides to absorb water in its structure. Basil seeds are easy to obtain and inexpensive. Therefore, they are suitable for producing drugs such as anticancer drugs [11], nanocomposites [12], etc. Yari et al. [1] studied the synthesis of hydrogel beads using BSM and sodium alginate (SA) to improve the encapsulation efficiency and optimal control of the release of metformin. They reported that increased amounts of BSM increased encapsulation efficacy. On the contrary, drug release ability decreased due to the stronger structure of the polymer. To prepare hydrogel beads, a biopolymer such as SA can rapidly form hydrogel beads with cations (for example, Zn^2+^, Ca^2+^, and Ba^2+^) at a neutral pH [13]. Hua et al. [14] reported that the encapsulation efficiency improved to 99.46% when using PVA mixed with SA at a ratio of 3:1 to form hydrogel beads. At a concentration of SA lower than this ratio, the bead form was imperfect.

Magnetic particles (MPs) have attracted a great deal of attention due to their potential for incorporation into hydrogel bead drug carriers [15]. MPs have a high surface area and low toxicity [16,17]. Furthermore, they can control the amount of drug released in different tissues in the body. This is because the human body has different pH levels in each organ; for example, the stomach has a pH of 1.2, while the intestine has a pH of 7.4 [2]. Supramaniam et al. [18] synthesized iron into magnetic nanoparticles in nanocellulose crystals (m-CNCs) to enhance mechanical strength and controlled release of ibuprofen from hydrogel tablets. A scanning electron microscope (SEM) micrograph revealed an increase in surface roughness with an increasing number of m-CNCs, resulting in a more porous structure and increased drug loading efficiency. The release of ibuprofen could be controlled at pH 1.2, 7.0, and 7.4. Moreover, the highest release, 3.2% ± 0.2%, and drug encapsulation, 38.2% ± 0.1%, were obtained from the sample with 3 wt% m-CNCs of SA solution. The pH-sensitive hydrogel was investigated for oral anticancer drug delivery based on magnetic particles [19]. The swelling ratio of the carboxymethylcellulose/polyacrylic acid/starch-modified Fe_3_O_4_ was affected by different contents of Fe_3_O_4_; i.e., the values increased at lower concentrations of Fe_3_O_4_, then decreased. Moreover, the results demonstrated the effect of pH on the swelling behavior of the hydrogel beads due to the shrinkage of the polymeric network under acidic conditions.

Our literature review indicated that using BSM and SA in optimal proportions can enhance drug encapsulation due to the stronger structure of the hydrogel beads and polymer chain, thus reducing drug release. An increase in MPs with a suitable content results in a rougher surface and more porosity, and can control the amount of drug release for body tissues with different pH values. Hence, to design a drug carrier with required properties, the design of the experiment (DOE) should analyze the impacts of individual components and their interactions [20]. Taguchi’s method is a well-known method for DOE, and is used to design the optimum mix proportions of polymer composites while requiring a minimal number of experiments [17]. The Taguchi method has been used to both optimize a single response and to determine the main factors responsible for the response. In the case of multi-response optimization problems, the Taguchi method can be coupled with Grey relational analysis to optimize multi-responses by converting the multi-responses to a single relational grade. Recently, the mix proportion of various biopolymer composites has successfully been designed and optimized using the Taguchi method and Grey relational analysis, for example, in the context of bioactive starch foam composites [21], bioactive film composites [22], and drug delivery systems [23].

The objective of this study was to optimize hydrogel beads as a drug carrier composed of BSM, SA, and MPs. The experiment in this study was designed using the Taguchi method and Grey relational analysis. The experimental design involved three factors, including BSM, SA, and MPs, with four levels of values to find the appropriate volume percentage of individual factors. The prepared samples had their mix proportions optimized for swelling, drug loading, drug release in different pH values, and antioxidant activity in different pH values. Kinetic drug release was investigated as well. The chemical and physical properties of the beads were examined using a scanning electron microscope (SEM), X-ray diffraction (XRD) analysis, and Fourier Transform Infrared Spectroscopy (FT-IR).

## 2. Results and Discussion

### 2.1. Morphology of the BSM/SA/MPs Hydrogel Beads

**Figure 1:**
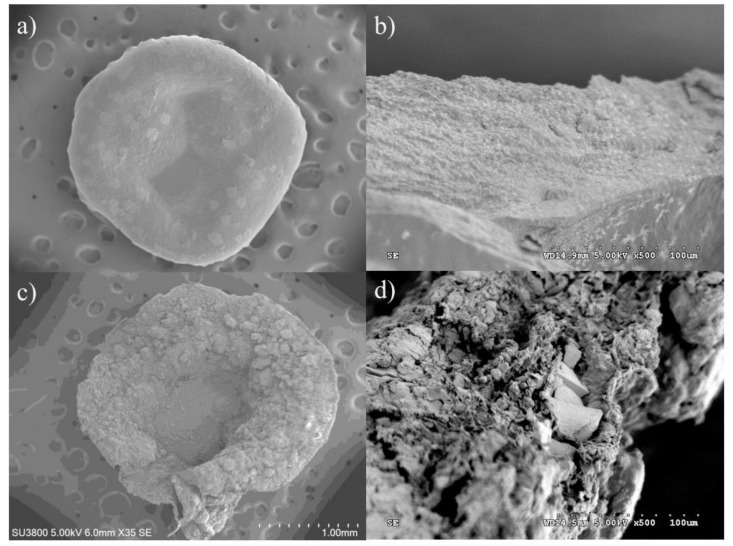
SEM images of hydrogel beads: (**a**) surface of sample from experimental design No. 4; (**b**) cross-section of sample from experimental design No. 4; (**c**) surface of sample from experimental design No. 16; and (**d**) cross-section of sample from experimental design No. 16.

SEM investigation was considered to observe the morphology of the BSM/SA/MPs hydrogel beads as they act as drug carriers, which is one of the most important factors affecting the administration route and drug release behavior. Figure 1 shows SEM micrographs of the cross-sectional hydrogel beads of the sample BSM/SA with and without MPs. Figure 1a,b shows the morphology of BSM/SA. A smooth surface and a porous structure with cavities were observed. Incorporation of MPs in the hydrogel beads led to a rougher surface and greater number of cavities inside compared to those without MPs, as shown in Figure 1c,d. The increase in cavities allowed the drug solution to be entrapped in the micro-environmental cavities and diffused into the hydrogel bead carrier more easily due to the capillary effect [1]. This structure was suitable for higher amounts of drug loading into hydrogel beads.

### 2.2. Chemical Functionality of the BSM/SA/MPs Hydrogel Beads

**Figure 2:**
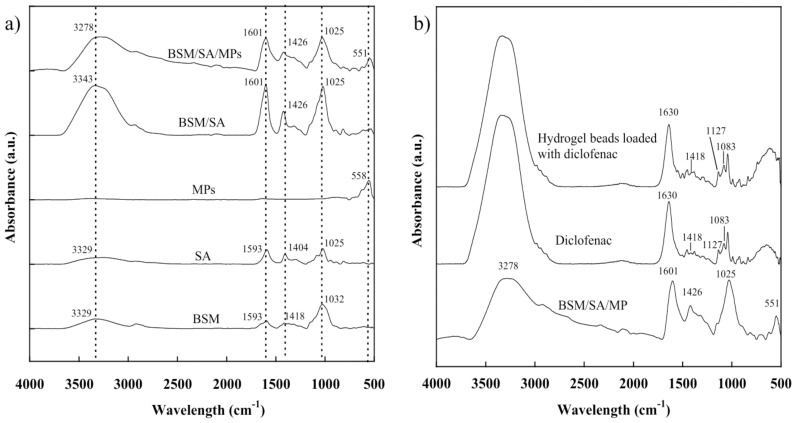
FTIR spectra of (**a**) chemical components and the BSM/SA/MPs hydrogel and (**b**) hydrogel beads loaded with diclofenac.

The chemical structures of the hydrogel beads were studied using the ATR-FTIR technique. From Figure 2a, for BSM the broad peaks appeared around 3000–3500 cm^−1^ due to vibrational stretching of O-H molecule and at 870–1150 cm^−1^, representing the C-O bond of the carbohydrate group. A small peak of BSM at 2850–3000 cm^−1^ related to the hydroxyl CH-bond oscillations, while the peak at 1593 cm^−1^ corresponded to C=C stretching for non-condensed alkanes [24]. The peak positions at 1025 cm^−1^ were due to CH stretching, 1404 and 1593 cm^−1^ were assigned to stretching of carboxyl groups, and the broad peak at 3000–3600 relating to the O-H group was observed in the SA [25]. The characteristic peak position of MPs was observed at a wavelength of 558 cm^−1^, indicating MP formation [19,26,27]. For the BSM/SA/MPs hydrogel beads, all characteristic peaks of BSM, SA, and MPs were observed and showed small changes in peak position, indicating physical interaction of the components [6,28,29].

The loading of diclofenac into hydrogel beads was confirmed by FTIR [30]. Figure 2b shows the characteristic peaks of diclofenac and hydrogel beads loaded with diclofenac. The principle FTIR spectra of diclofenac appeared at 1630 cm^−1^ (C=O stretching vibration of the carboxyl group), 1418 cm^−1^ (C=C vibration of aromatic ring), 1127 cm^−1^, and 1083 cm^−1^ (C-H deformation vibration of aromatic ring), and band in the range of 3000–3300 cm^−1^ (N-H vibration) [31]. After loading diclofenac into hydrogel beads, all the principal characteristic peaks of diclofenac were observed in the hydrogel beads.

### 2.3. XRD Pattern of the BSM/SA/MPs Hydrogel Beads

**Figure 3:**
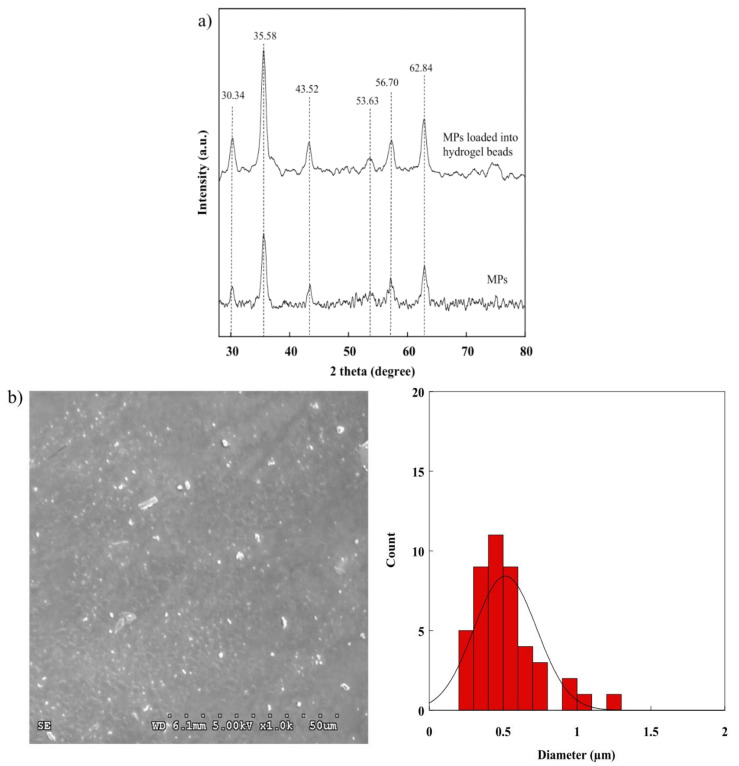
(**a**) XRD pattern of MPs and (**b**) SEM images of MPs.

There are several methods of synthesizing MPs. In this research, co-precipitation was used, as it is a simple and easy technique. The successful preparation of MPs is evidenced by XRD observation. As shown in Figure 3a, 2θ peaks were found at 30.34°, 35.58°, 43.52°, 53.63°, 56.70°, and 62.84°, and were assigned to (220), (311), (222), (422), (511), and (440) corresponding to the standard XRD format of International Center for Diffraction Data (ICDD) No. 019-0629 of Fe_3_O_4_ with a cubic crystal structure [32,33,34]. After adding MPs into hydrogel beads, the peaks of other iron oxides could not be observed. This observation confirmed that the MPs were successfully incorporated into hydrogel beads without oxidization. Figure 3b shows a particle-sized distribution of MPs in the range of 0.42–1.01 µm.

### 2.4. Swelling Study of the BSM/SA/MPs Hydrogel Beads

**Figure 4:**
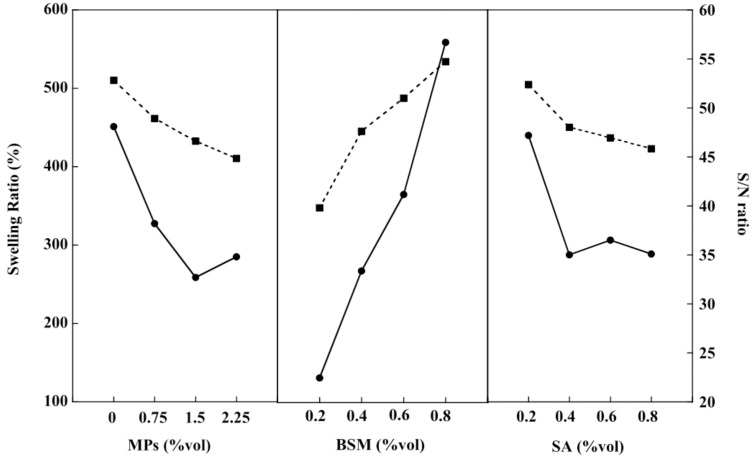
Swelling (–●–) and S/N (–■–) ratios of the BSM/SA/MPs hydrogel beads.

The swelling property is one most important properties for drug delivery, as it affects the release rate of the drug to the medium [13]. Consequently, the effects of various compositions of BSM, SA, and MPs on the swelling property of the hydrogel beads were investigated in order to better understand each component’s role in the release mechanism. Upon immersion, all of the hydrogels took up water, and the dimensions of the hydrogels changed due to the interaction of water molecules with the functional ionic groups of hydrogel beads and electrostatic repulsion between charges on a polymer chain, thus leading to an increase in swelling [6]. Figure 4 shows that the swelling value of the BSM/SA/MPs hydrogel beads increased sharply with increasing BSM concentration. This was attributable to the fact that BSM exhibits hydrophilicity due to its heteropolysaccharide structures of glucomannan and xylan, which have large amounts of hydroxyl groups [35]. In comparison, the swelling values of BSM/SA/MPs were in the range of 130–560%, higher than those reported in SA/BSM beads, which had values in the range of 150–250% [1]. The hydrophilic property of materials enhances the swelling efficiency, resulting in good diffusion of drugs from hydrogel beads into media solution. In contrast, the swelling value tended to decrease as the percentage of MPs and SA increased. The intermolecular chain of SA and BSM might create a tight structure, whereas MPs could create interaction with the polymer matrix and interaction between magnetic particles [36,37]. Moreover, this was related to osmotic pressure and an increased intermolecular electrostatic repulsion force, resulting in water penetrating into the internal structure of the hydrogel [38]. A high S/N ratio indicates high swelling of hydrogel beads. The S/N ratio values were in good agreement with the larger-the-better criterion. The amount of BSM is the main factor affecting the hydrogel beads’ swelling properties, with a contribution of 68.17%, as summarized in Table 1. The optimum composition of the BSM/SA/MPs hydrogel providing the highest swelling ratio was at 0 %vol of MPs, 0.8 %vol of BMS, and 0.2 %vol of SA. The multi-linear regression technique was applied to obtain the relationship between the swelling and the composition of hydrogel beads, as expressed in Equation (1):

(1) $$Swelling=-0.01X_{1}+0.01X_{2}+0X_{3}+1.15$$

where X_1_ is MPs (%vol), X_2_ is BSM (%vol), and X_3_ is SA (%vol).

### 2.5. Drug Loading of the BSM/SA/MPs Hydrogel Beads

**Figure 5:**
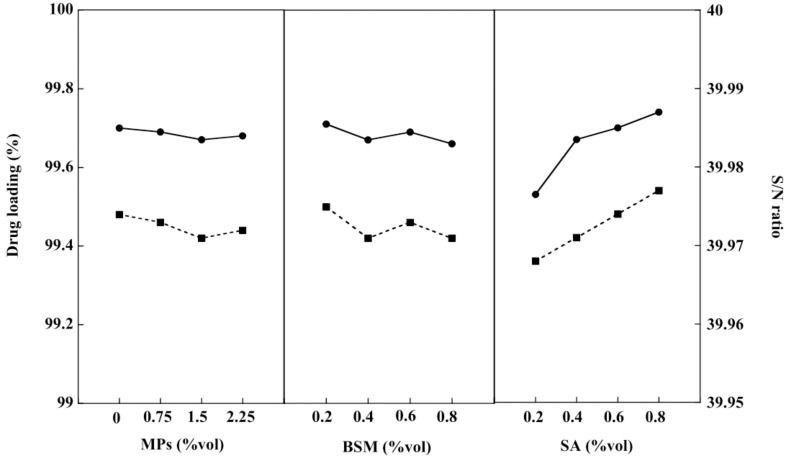
Drug loading (–●–) and S/N ratio (–■–) of the BSM/SA/MPs hydrogel beads.

Consistent with Ecke et al., this study used diclofenac, a drug for relief of inflammation, pain, and intumescence, as a drug model to simulate drug transport and investigate drug loading of the BSM/SA/MPs hydrogel beads [39]. Drug loading of the hydrogel beads tended to decrease with increasing amounts of BSM and MPs, whereas the values gradually increased with SA content, as shown in Figure 5. This was attributed to the hydrophilicity of SA as a polymer matrix that facilitated the formation of hydrogel beads. Moreover, the structure of SA, containing a carboxylate group (–COO–) exhibited, an electrical attraction to the drug, thus allowing the drug to be contained and causing greater drug loading [40]. The increase of BSM enhanced the connection between BSM and SA. This phenomenon might have reduced the cavities inside the beads [1,36]. Similarly, the swelling property of hydrogel beads decreased when MP content increased. Mahdavinia et al. [37] suggested that adding MPs to hydrogel beads could create interaction with the polymer matrix, decreasing hydrophilicity. From Table 1, it can be seen that SA was the main factor, accounting for 61.91% of the drug loading value. The composition of BSM/SA/MPs providing the maximum drug loading was at 0 %vol of MPs, 0.2 %vol of BSM, and 0.8 %vol of SA. The value of drug loading of the BSM/SA/MPs was in the range of 99.3 to 99.7%, which was higher than that reported in micelles/sodium alginate composite gel beads [41]. A multi-linear regression analysis was used to formulate the relationship between drug loading and hydrogel bead components, as expressed in Equation (2):

(2) $$Drug\;loading=6.61X_{1}+2.67X_{2}+30.04X_{3}-3916.691$$

where X_1_ is MPs (%vol), X_2_ is BSM (%vol), and X_3_ is SA (%vol).

### 2.6. In Vitro Drug Release of the BSM/SA/MPs Hydrogel Beads

**Figure 6:**
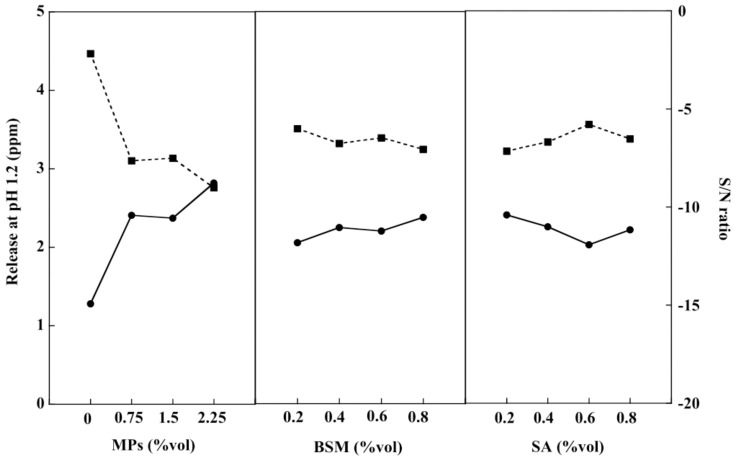
Drug release at pH 1.2 (–●–) and S/N ratio (–■–) of the BSM/SA/MPs hydrogel beads.

Drug release studies of the BSM/SA/MPs hydrogel beads were conducted in acid and neutral PBS solution at 37 °C to investigate the effect of pH on the drug release rate, similar to Yin et al. [42]. The PBS solution at pH 1.2 was prepared to simulate gastric fluid in the human stomach, and the release behavior of diclofenac was investigated [43]. As shown in Figure 6, it was found that the release of the drug from the BSM/SA/MPs hydrogel beads slightly increased with BSM. The drastic increase of drug release from the sample with a large number of MPs was observed due to the repulsion force between positive charges of MPs and the drug [44]. The diclofenac release tended to decrease when SA increased. The carboxylic group (–COO–) in SA exhibited a negative charge and formed an attractive force to the positive charge of the drug [45]. The results of drug release in pH 1.2 corresponded to the S/N ratio with the smaller-the-better criteria. Table 1 shows that MPs influenced drug release at pH 1.2, accounting for 87.75%. The most suitable composition of the BSM/SA/MPs hydrogel beads providing the lowest release rate of diclofenac in PBS at pH 1.2 was at 0 %vol of MPs, 0.2 %vol of BSM, and 0.6 %vol of SA.

The swelling ratio of the hydrogel beads at pH 1.2 after 3 h of incubation was less than 0.6% due to the pKa of SA being about 3.2 [46], and the formation of insoluble alginic acid resulted in protonation of carboxyl groups (–COOH) in SA. However, ionization of the carboxyl groups of SA (–COO–) took place at pH 7.4. Due to osmotic pressure and increased intermolecular electrostatic repulsion forces, water penetration occurred on the internal structure of the hydrogel [1,41].

**Figure 7:**
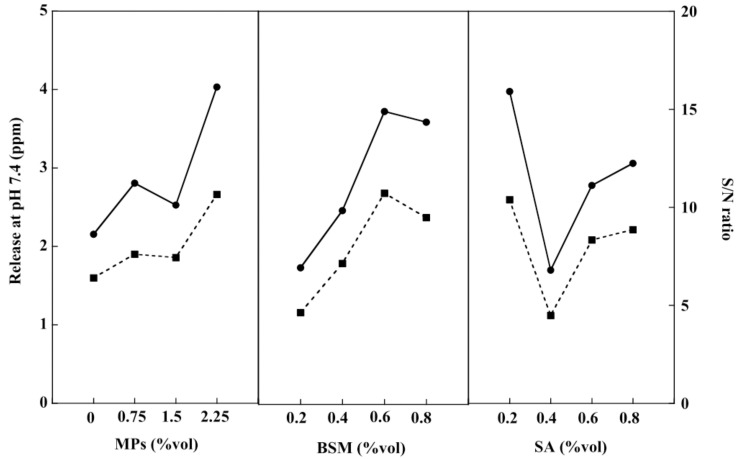
Drug release at pH 7.4 (–●–) and S/N ratio (–■–) of the BSM/SA/MPs hydrogel beads.

A PBS solution at pH 7.4 was prepared to simulate human intestinal fluid and the release rate of the drug from the BSM/SA/MPs hydrogel bead was studied. From Figure 7, it can be seen that the release of the drug tended to increase with a greater amount of BSM and MPs. However, an increase in SA content resulted in a reduction in drug release. This was attributed to the carboxylic groups of SA being neutralized at pH 7.4, leading to the repulsion force acting on the drug, then the release rate of the drug increasing with SA content. Moreover, it could be related to the opened pores caused by ionic repulsion of the constituted ions, which can be formed via ionization of carboxyl groups of BSM and hydroxyl groups on the SA chain [6]. BSM and SA were the major components affecting the release of the drug at pH 7.4. The hydrogel beads containing 2.25 %vol of MPs, 0.6 %vol of BSM, and 0.2 %vol of SA had the most suitable composition, providing the highest drug release at pH 7.4. Similarly, per multi-linear regression analysis drug release at pH 1.2 and pH 7.4 was formulated as a function of hydrogel bead components, as expressed in Equations (3) and (4), respectively:

(3) $$Drug\;release\;at\;pH1.2=-5.34X_{1}+29.55X_{2}-8.99X_{3}-31.25$$

(4) $$Drug\;release\;at\;pH7.4=0.73X_{1}+0.9X_{2}+0.06X_{3}-31.25$$

where X_1_ is MPs (%vol), X_2_ is BSM (%vol), and X_3_ is SA (%vol).

### 2.7. Release of Antioxidants from Hydrogel Beads

**Figure 8:**
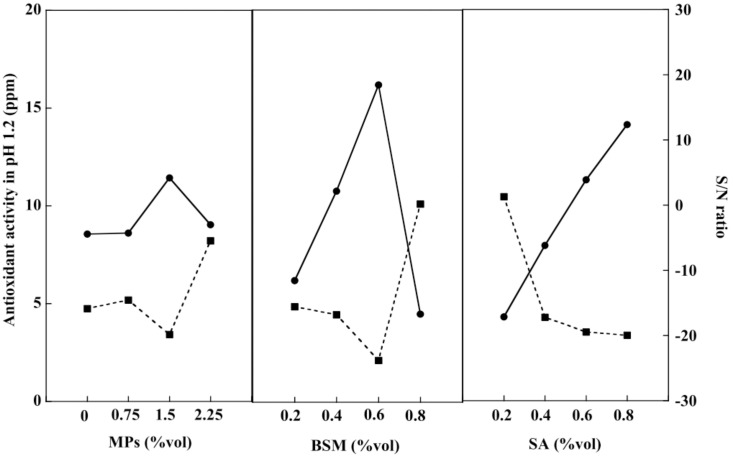
Release of antioxidant at pH 1.2 (–●–) and S/N ratio (–■–) of the BSM/SA/MPs hydrogel beads.

An antioxidant is an important chemical compound for humans because it helps the immune system work more efficiently. The human body eliminates waste products in the form of free radicals. These cause damage to the immune system, resulting in various diseases including heart disease, vascular disease, and cancer [47]. BSM contains nutrients such as vitamin E, an important substance that helps the body produce free antioxidants [48]. Consequently, the antioxidant activities of the BSM/SA/MPs hydrogel beads were studied at pH 1.2 and pH 7.4. Figure 8 shows that the release of antioxidants at pH 1.2 tended to increase with the increasing amount of all components in the hydrogel. BSM was the main contributor to the release of antioxidants at pH 1.2, accounting for 41.31%, followed by SA with a 27.26% contribution. According to the smaller-the-better criterion, the best composition providing the least antioxidant activity at pH 1.2 was at 0 %vol of MPs, 0.8 %vol of BSM, and 0.2 %vol of SA.

**Figure 9:**
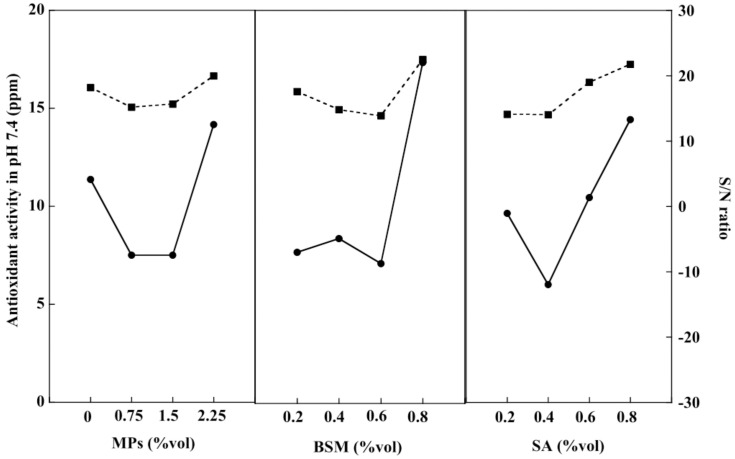
Release of antioxidant at pH 7.4 (–●–) and S/N ratio (–■–) of the BSM/SA/MPs hydrogel beads.

Figure 9 shows the antioxidant activity of the hydrogel beads at pH 7.4. The value tended to increase with an increasing amount of BSM, SA, and MPs. BSM was the major factor, contributing about 26.73% to the antioxidant activity, whereas SA and MPs contributed almost equally to the release of antioxidants. The relationship of antioxidant release was obtained by the regression method, as expressed in Equation (5):

(5) $$Values\;of\;antioxidant\;release=-0.5X_{1}+0.3X_{2}+0.31X_{3}+1.33$$

where X_1_ is MPs (%vol), X_2_ is BSM (%vol), and X_3_ is SA (%vol).

### 2.8. Optimization of the Hydrogel Bead Compositions Using Grey Relational Analysis

Grey relational analysis was used in conjunction with Taguchi’s experimental method in this study. The optimal composition of the BSM/SA/MPs hydrogel beads with the best overall properties was determined as shown in Table S1. Grey relational coefficient calculation properties for the L-16 comparability sequence included drug release at pH 1.2 and 7.4, antioxidant release at pH 1.2 and 7.4, drug loading, and swelling of the hydrogel beads. The highest concentration of Grey relational grade was 0.704, which contained 2.25 %vol MPs, 0.8 %vol BSM, and 0.2 %vol SA. The maximum value of the response results of the Grey relational analysis indicates the suitability of each factor affecting overall properties [49].

A confirmation test was conducted to determine the accuracy of the optimal combination. The predicted optimal properties of the BSM/SA/MPs hydrogel beads were calculated as shown in Equation (6):

(6) $$\hat{\gamma }=\gamma_{m}+\sum_{i=1}^{q}\left(\gamma_{i}-\gamma_{m}\right)$$

where $\gamma_{m}$ is the total mean of Grey relational grade, $\gamma_{i}$ is the mean of the Grey relational grade at the optimal level, and *q* is a number matching the parameters that significantly affected multiple performance characteristics. As seen in Table 2, the experimental value agreed with the predicted value.

### 2.9. Effect of pH on Drug Release of the BSM/SA/MPs Hydrogel Beads

**Figure 10:**
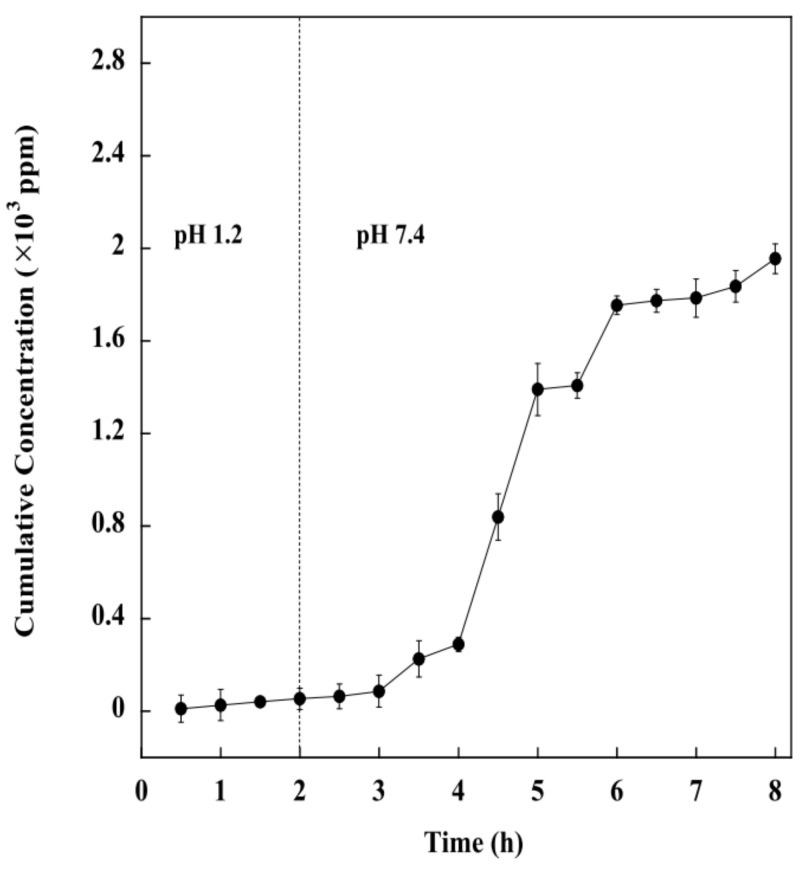
Release of drug from hydrogel at pH 1.2 and 7.4.

Regarding the most suitable composition, the BSM/SA/MPs hydrogel beads containing 2.25 %vol of MPs, 0.8 %vol of BSM, and 0.2 %vol of SA were selected to further study the kinetic release of drug from the hydrogel beads. The pH environment in the gastrointestinal tract varies from acidic to slightly alkaline. Therefore, the kinetic release study was carried out at pH 1.2 and again at pH 7.4. Figure 10 shows that the cumulative release amount of diclofenac from the BSM/SA/MPs hydrogel beads was significantly influenced by pH value. It was demonstrated that the drug release at pH 7.4 was higher than at pH 1.2. The drug release in an acidic medium slightly increased within 2 h and drastically increased when the hydrogel was transferred into a medium with pH 7.4. This can be explained by the minimum swelling of the hydrogel beads under acidic conditions. It was reported that the alginate polymer chain was catalytically hydrolyzed under acidic conditions to low molecular alginic acid [36]. It was additionally related to the shrinkage of the polymeric network due to the physical crosslinker generated from H-bonding among protonation of carboxylic group of the polymer under acidic conditions [19], and moreover because the carboxylate group (–COO–) of SA forms an attraction force with the drug, resulting in slower release of drug by the hydrogel beads [50,51,52]. A rapid drug release rate was observed at pH 7.4 because of the deprotonation of the carboxylate group of SA, resulting in an electrostatic repulsion force between the carboxylate group and the SA. Therefore, the drug was released rapidly. In the final stage, small molecules of alginic acid cannot retain the hydrate structure, and the hydrogel beads start to lose their overall structure. Consequently, the BSM/SA/MPs hydrogel beads showed changes in swelling properties in response to the pH of the medium, and can thus be used as a pH-responsive drug delivery system.

Mathematical modeling of drug release kinetics provides a basis for the study of mass transport mechanisms that are involved in the control of drug release. There are several comprehensive reviews on mathematical modeling for bioerodible polymeric delivery systems, dissolution-controlled drug delivery systems, microsphere delivery systems, and hydrogel networks. In general, diffusion, erosion, and degradation are the most important mechanisms for drug transport from polymeric matrices [53]. The release kinetics of diclofenac from the BSM/SA/MPs hydrogel beads into PBS at pH 7.4 were studied using four different release mathematical models, zero-order, hirst-order, Higuchi, and Korsmeyer–Peppas. For the kinetic study, the following relationships were plotted: %cumulative drug release vs. time (zero-order kinetic model); % log cumulative drug remaining vs. time (first-order kinetic model); %cumulative drug release vs. square root of time (Higuchi model); % log cumulative drug release vs. log time (Korsmeyer–Peppas model). The zero-order behavior reflects the release of the drug at fixed rates at all investigated intervals and involves no effect on its concentration as a loaded drug. The first-order behavior involves a significant role of drug concentration during its release from the carriers. The Higuchi kinetic behavior demonstrates the diffusion of the drug as the release mechanism considering the reported assumptions of the model. The obtained kinetic curves and parameters are presented in Figure S1 and Table 3. The optimal fitting model was determined based on the highest correlation coefficient (R^2^) obtained from regression analysis.

It was found that Korsmeyer–Peppas was the model that best agreed with kinetic release of the model drug in PBS at PH 7.4, providing the highest R^2^ value of 0.989 ± 0.097. From the Korsmeyer–Peppas model, the release value *n* for the hydrogel beads was lower than 0.5. The mathematical result indicated that the levels of diclofenac release rate from the hydrogel during the release time are probably related to drug diffusion near the hydrogel surfaces, and further revealed that the release of drug from the hydrogel took place through the quasi-Fickian diffusion mechanism, which is associated with a concentration gradient, diffusion distance, as well as with the degree of swelling.

## 3. Conclusions

This study developed BSM/SA/MPs hydrogel beads by optimizing their components. The experiment was designed using a Taguchi experimental design in combination with Grey relational analysis. The results indicated that BSM contributed most to the swelling behavior of the hydrogel beads, while drug loading was most affected by the addition of SA. The hydrogel beads were demonstrated to be pH sensitive to diclofenac release rate, with MPs being the principal components affecting the release rate in an acidic medium. In contrast, BSM and SA influenced the release rate in a neutral or mildly alkaline medium. The BSM/SA/MPs hydrogel beads exhibited antioxidant activity in media with different pH values, with BSM the major contributing factor. Using Grey relational analysis, the composition that provided the best properties in terms of swelling ratio, drug loading, release of diclofenac at pH 1.2 and 7.4 and antioxidant activity at pH 1.2 and 7.4 consisted of 2.25% MPs, 0.8% BSM, and 0.2% SA. It can be concluded that BSM/SA/MPs could be applied for pH sensitive intestine-specific drug delivery systems.

## 4. Materials and Methods 

### 4.1. Materials

SA (91%–106%, LOBA CHEMIV PVT. Ltd., Mumbai, India), sodium hydroxide (99%, FW 40 RCI LabScan Limited, Bangkok, Thailand), Iron (III) chloride hexahydrate (97–102%, FeCl_3_·6H_2_O; MW 270.32, KEMAUS, New South Wales, Australia), Iron (II) sulfate heptahydrate (FeSO_4_·7H_2_O; 99.5–104.5%, MW 278.02, KEMAUS, New South Wales, Australia), Calcium chloride, hydrochloric acid (97%, Mw. 110.99, KEMAUS, New South Wales, Australia), dipotassium phosphate (K_2_HPO_4_; 99%, MW 174.18, KEMAUS, New South Wales, Australia), Potassium dihydrogen phosphate (KH_2_PO_4_; 99.5%, MW 136.09, RCI LabScan Limited, Bangkok, Thailand), 2,2-diphenyl-2-picryl-hydrazyl (DPPH) (MW. 394.32, Sigma-Aldrich, Singapore), and PVA with a DP of 1700–1800 were supplied by RCI Labscan Limited, Bangkok, Thailand. Diclofenac sodium was purchased from Lincoln Parenterals Pvt. Ltd, Gujarat, India.

### 4.2. Preparation of BSM

Basil seeds were soaked in deionized water (DI water) at a mass ratio of 1:50 for 2 h. The basil seeds were then spun with an overhead stirrer and heated to 70 °C for 12 h, using reflux equipment to prevent the water from evaporating. The BSM solution was centrifuged at 7000 rpm for 15 min, then BSM was filtered from the water. The BSM was poured into a petri dish and incubated at a temperature of 60 °C for 12 h to obtain dried BSM powder.

### 4.3. Preparation of MPs

FeCl_3_·6H_2_O was stirred into 125 mL DI water at 550 rpm at a temperature of 90 °C for 10 min. Then, 2.7 g of FeSO_4_·7H_2_O was added to the mixture and further stirred for 10 min. NaOH solution was prepared separately by dissolving 10 g of NaOH in 40 mL DI water. NaOH solution was added to FeSO_4_·7H_2_O solution. The dark precipitate was obtained and heated at a temperature of 90 °C for 1 h. After being cooled to room temperature, it was filtered, washed with DI water until pH 7.0–7.5 was reached, and dried at room temperature for 6 h.

### 4.4. Hydrogel Bead Preparation

Dried BSM was added to 40 mL of DI water and stirred for 2 h. The BMS/SA was prepared by adding SA solution to the basil seed mixture. The iron particles were added to obtain BSM/SA/MPs. The BSM/SA/MPs were added dropwise into the prepared CaCl_2_ solution under continuous stirring. The resulting hydrogel beads were then rinsed several times and dried at a temperature of 40 °C for 6 h. The overall steps in hydrogel bead preparation are exhibited in Scheme S1.

The sol fraction of hydrogel beads was analyzed according to Nawaz et al. [54]. The sol part implied the unreacted portion of the formulation in the hydrogel. Based on previous reports, the sol fraction was reported to be 5–25% [51,54,55,56,57]. The sol fraction of the sixteen formulations of hydrogel beads was 7.07–49.55%, as shown in Supplemental Table S2.

### 4.5. Characterizations

The FTIR of hydrogel beads was carried out using a Bruker TENSOR27 (Billerica, United State), FTIR. The spectrum was analyzed at 4000–600 cm^−1^ with a resolution of 2 cm^−1^ for 64 scans.

The X-ray diffraction (XRD) of Fe_3_O_4_ was tested using an EMPYREAN X-ray diffractometer (Malvern, UK) provided with a Cu Kα radiation source operated at 45 kV and 40 mA. The sample was scanned at diffraction angles of 20° ≤ 22θ ≤ 80°.

The structures of hydrogel beads were observed using scanning electron microscopy (SEM) (Hitachi SU-4800, Tokyo, Japan). The field emission scanning electron microscope was accelerated at a voltage of 3.0 kV and an emission current of 10 mA. The surfaces of the samples were sputter-coated with gold before measurement.

Kinetic drug release from the hydrogel beads was studied. The prepared hydrogel beads were loaded into a dialysis bag and suspended in 0.01 M of PBS at a temperature of 37 °C under magnetic stirring at 80 rpm. The release kinetics analysis was carried out in PBS at pH 1.2 for 2 h (as the average gastric empty time is about 2 h), and again at pH 7.4 for a predetermined time. The PBS solution was taken every 30 min for 3 h, and the same amount of fresh PBS was added to the system. The sample was then measured using a UV-Visible spectrophotometer (Agilent Cary 60, Santa Clara, CA, USA) at 222 nm.

The drug release mechanism from the hydrogel was studied by fitting the experimental result using four different release kinetic models, zero order, first order, Higuchi, and Korsmeyer–Peppas. Their equations are presented in Equations (7)–(10). These models were selected because they are widely used to explain drug release from polymers when several types of release mechanisms are involved. The model with the highest R^2^ value was the most suitable model to describe the released kinetic of the hydrogel beads.

(7) $$\mathrm{Zero}\;\mathrm{order}\;\mathrm{model}:\;\;C=k_{o}\;t,\;$$

where C is the amount of diclofenac released at time t, k_0_ is the zero-order rate constant, and t is the time.

(8) $$\mathrm{First}-\mathrm{order}\;\mathrm{model}:\;\;\;\ln C_{t}=\ln C_{o}-k_{1}\;t$$

where C is the amount of diclofenac released at time t, k_1_ is the zero-order rate constant, and t is the time.

(9) $$\mathrm{Higuchi}\;\mathrm{model}:\;{M=\mathrm{kt}}^{\left(1/2\right)}$$

where M is the diclofenac release amount at time t and k is the diffusion rate constant.

(10) $$\mathrm{Korsmeyer}–\mathrm{Peppas}\;\mathrm{model}:\;\frac{M_{t}}{M_{\infty }}=kt^{n},$$

where M_t_ is the amount of diclofenac released at time t, M_∞_ represents the amount of diclofenac released at equilibrium state, *n* is the release exponent, and k is the constant value of the drug–composite system.

The value of n is determined to identify the mechanism:

*n* < 0.5; quasi-Fickian diffusion

*n* = 0.5; normal Fickian diffusion

*n* = 0.5–1.0; non-Fickian or preposterous transport

*n* = 1.0; Case II diffusion.

The DPPH radical scavenging activity of nanocomposite film was studied using the method proposed by Brand Williams et al. [58]. The hydrogel beads were crushed and mixed with ethanol solution, and the sample was then ultrasonicated at 500 W at a temperature of 37 °C for 30 min. The ethanol solution mixed with 0.4 mM methanolic DPPH was blended with the samples under a dark atmosphere and incubated for 30 min. The absorbance of the blend was assessed utilizing UV-spectrometer at a wavelength of 517 nm. The films’ DPPH free radical scavenging activity was determined using Equation (11).

(11) $$DPPH\;free\;radical\;scavenging\;activity\;(\%)=\frac{Abs_{DPPH}-Abs_{Extract}}{Abs_{DPPH}}$$

where ${\mathrm{Abs}}_{\mathrm{DPPH}}$ is the absorbance at 517 nm of the DPPH solution and ${\mathrm{Abs}}_{\mathrm{Extract}}$ represents the absorbance of the extracted sample.

Swelling of the hydrogel beads was measured in DI water at pH levels of 1.2 and 7.4 for 24 h. The swelling (%) was calculated according to Equation (12)

(12) $$Swelling\;(\%)=\frac{W_{s}-W_{i}}{W_{i}}\times 100$$

where W_s_ and W_i_ represent the weight of the hydrogel beads after swelling and before swelling, respectively.

The percentage of drug loading on the hydrogel beads was determined based on Equation (13)

(13) $$Drug\;loading\;(\%)=\frac{C_{i}-C_{s}}{C_{i}}\times 100$$

where C_i_ represents the initial drug concentration and C_s_ is the concentration of free drug in the supernatant [59].

The Taguchi method, containing three factors and four levels, was used to optimize the conditions of the properties of hydrogel beads for drug delivery in terms of single response and multiple responses. The conditions and their variation levels are shown in Table 4 and Table 5. The signal to noise ratio (S/N) was used to evaluate the effect of each parameter level for single response optimization with the help of ANOVA.

The S/N ratios were classified into three classes: (1) nominal-the-better, (2) smaller–the-better, and (3) larger-the-better, which were applied for optimization [60]. In this study, all responses, including drug release at pH 1.2 and release of antioxidants at pH 1.2, were minimized, corresponding to “smaller-the-better.” Swelling (%), drug loading (%), drug release at pH 7.4, and release of antioxidants at pH 7.4 were maximized, corresponding to “larger-the-better”, and the S/N ratio was analyzed based on Equations (14) and (15), respectively.

(14) $$(S/N)\;=-10\log\left[1/R\sum_{f=1}^{R}y_{i}^{2}\right]\;$$

(15) $$(S/N)\;=-10\log\left[1/R\sum_{f=1}^{R}1/y_{i}^{2}\right]\;$$

where *R* is the number of all data points and *y_i_* is the value of *i*th data point.

Grey relational analysis was used to convert multiple optimization responses into a single response optimization by calculating S/N ratios. The obtained results from the Taguchi method were then calculated to determine the highest overall Grey relational, which represents the optimal parametric combination. Prior to Grey relational analysis, data preprocessing is normally required to transfer the original sequence to a comparable sequence that is normalized within the range of zero to one [61]. The reference sequence and comparable sequence can be denoted by *x_o_*(*k*) and *x_i_*(*k*) for *i* = 1, 2, …, m; *k* = 1, 2, …, n, respectively, where m is the total number of experiments to be considered and n is the total number of observation data. The appropriate equation for this normalization depends on the type of the quality characteristic. In this work, smaller-the-better and larger-the-better quality characteristics were applied to normalize all the responses, as expressed in Equations (16) and (17), respectively

(16) $$x_{i}\left(k\right)=\frac{max\;y_{i}\left(k\right)-y_{i}\left(k\right)}{max\;y_{i}\left(k\right)-min\;y_{i}\left(k\right)}$$

(17) $$x_{i}\left(k\right)=\frac{y_{i}\left(k\right)-min\;y_{i}\left(k\right)}{max\;y_{i}\left(k\right)-min\;y_{i}\left(k\right)}$$

where *x_i_(k)* is the value after Grey relational generation, min *y_i_(k)* is the smallest value of *y_i_(k)* for kth response, and *y_i_(k)* max *y_i_(k)* is the largest value of *y_i_(k)* for kth response.

The Grey relational coefficient can be calculated using Equation (18)

(18) $$\gamma (x_{o}^{*}(k),x_{i}^{*}(k)\;)=\frac{\;\Delta_{\min}+\zeta \Delta_{\max}}{\Delta_{0i}(k)+\zeta \Delta_{\max}}$$

$$0\;<\;\gamma (x_{o}^{*}\left(k\right),x_{i}^{*}\left(k\right)\leq \;1$$

where

$$\Delta_{0i}(k)=\left|x_{0}^{*}(k)-x_{i}^{*}(k)\right|$$

$$\Delta_{\max}=\overset{\max.}{\forall j\in i}\overset{\max.}{\forall k}\left|x_{0}^{*}(k)-x_{j}^{*}(k)\right|$$

$$\Delta_{\min}=\overset{\min.}{\forall j\in i}\overset{\min.}{\forall k}\left|x_{0}^{*}(k)-x_{j}^{*}(k)\right|$$

$x_{0}^{*}$ is the reference sequence, $x_{i}^{*}$ is given sequence, $x_{j}^{*}$ is comparison sequence and ζ is the distinguishing coefficient, $\zeta \in \left[0,1\right]$. 

If all process parameters have equal weighting, ζ is set to be 0.5. The Grey relational grade is the average of all Grey relational coefficients determined using Equation (19)

(19) $${\gamma (x}_{o}^{*}{,x}_{i}^{*})=\frac{1}{n}\sum_{k=1}^{R}\beta_{k}{\gamma (x}_{o}^{*}{(k),x}_{i}^{*}(k))$$

Finally, the optimal condition of the BSM/SA/MPs hydrogel beads is the level corresponding to the highest value of the average Grey relational grade of each factor. Table 4 and Table 5 show the factors and their levels, including the Taguchi experimental design.

## Figures and Tables

**Table 1:** ANOVA analysis results.

Factor	DF ^a^	SS ^b^	MS ^c^	F-Value	*p*-Value	Contribution (%)
Swelling						
MPs	3	87,231.00	29,077	5.97	0.031	15.33
BSM	3	387,831.00	129,277	26.56	0.001	68.17
SA	3	64,681.00	21,560	4.43	0.058	11.37
Error	6	29,200.00	4867	-	-	5.13
Total	15	568,944.00	-	-	-	100.00
Drug loading						
MPs	3	0.0014	0.0005	0.29	0.830	3.31
BSM	3	0.0051	0.0017	1.07	0.429	12.14
SA	3	0.0259	0.0086	5.47	0.038	61.91
Error	6	0.0095	0.0016	-	-	22.64
Total	15	0.0418	-	-	-	100.00
Release rate at pH 1.2						
MPs	3	5.2066	1.7355	49.41	0.000	87.95
BSM	3	0.2119	0.0706	2.01	0.214	3.58
SA	3	0.2906	0.0969	2.76	0.134	4.91
Error	6	0.2108	0.0351	-	-	3.56
Total	15	5.9199	-	-	-	100.00
Release rate at pH 7.4						
MPs	3	7.9350	2.6450	0.86	0.513	16.58
BSM	3	10.8010	3.6000	1.16	0.398	22.56
SA	3	10.5900	3.5300	1.14	0.405	22.12
Error	6	18.5460	3.0910	-	-	38.74
Total	15	47.8720	-	-	-	100.00
Antioxidant activity at pH 1.2						
MPs	3	22.4500	7.4850	0.20	0.894	2.83
BSM	3	328.2000	109.4010	2.89	0.125	41.31
SA	3	216.6000	72.2010	1.91	0.230	27.26
Error	6	227.2300	37.8710	-	-	28.60
Total	15	794.4800	-	-	-	100.00
Antioxidant activity at pH 7.4						
MPs	3	126.9000	42.3000	0.510	0.690	12.10
BSM	3	280.3000	93.4200	1.12	0.411	26.73
SA	3	143.0000	47.6700	0.57	0.653	13.63
Error	6	498.7000	83.1100	-	-	47.55
Total	15	1048.8000	-	-	-	100.00

^a^ Degree of freedom, ^b^ Sum of squares, ^c^ Mean square.

**Table 2:** Results of confirmation experiment.

	Predicted	Experiment
Grey relational grade	0.720	0.584

**Table 3:** The fitting data of model drug release obtained with different mathematical models.

Models	Parameters	PBS at pH 7.4
Zero order	*k*_0_	387.400 ± 0.092
	R^2^	0.957 ± 0.076
First order	*k*_1_	1.106 ± 0.081
	R^2^	0.783 ± 0.109
Higuchi	*k_H_*	0.495 ± 0.095
	R^2^	0.875 ± 0.103
Kormeyer-Peppas	*k_KP_*	0.215 ± 0.087
	*n*	0.207 ± 0.098
	R^2^	0.989 ± 0.097

**Table 4:** Factors and their levels.

Parameter	Level 1	Level 2	Level 3	Level 4
BSM (%vol)	0.2	0.4	0.6	0.8
SA (%vol)	0.2	0.4	0.6	0.8
MP (%vol)	0.0	0.75	1.50	2.25

**Table 5:** Taguchi experimental design.

No.	Composition (%vol)		
	BSM	SA	MPs
1	0.2	0.2	0.0
2	0.4	0.4	0.0
3	0.6	0.6	0.0
4	0.8	0.8	0.0
5	0.4	0.2	0.75
6	0.2	0.4	0.75
7	0.8	0.6	0.75
8	0.6	0.8	0.75
9	0.6	0.2	1.50
10	0.8	0.4	1.50
11	0.2	0.6	1.50
12	0.4	0.8	1.50
13	0.8	0.2	2.25
14	0.6	0.4	2.25
15	0.4	0.6	2.25
16	0.2	0.8	2.25

## Supplementary Materials

The following supporting information can be downloaded at: https://www.mdpi.com/article/10.3390/gels8050274/s1. Scheme S1. Overall steps for hydrogel bead preparation; Table S1. The properties for Grey relational coefficient calculation for the L16 comparability sequences; Table S2. Sol fraction of the hydrogel beads; Figure S1 shows the release profiles obtained using the four mathematical models.

## References

[1] Yari K., Akbari I., Yazdi S.A.V. (2020). Development and evaluation of sodium alginate-basil seeds mucilage beads as a suitable carrier for controlled release of metformin. Int. J. Biol. Macromol..

[2] Rayegan A., Allafchian A., Abdolhosseini Sarsari I., Kameli P. (2018). Synthesis and characterization of basil seed mucilage coated Fe(3)O(4) magnetic nanoparticles as a drug carrier for the controlled delivery of cephalexin. Int. J. Biol. Macromol..

[3] Singh A.K., Nutan B., Raval I.H., Jewrajka S.K. (2018). Self-assembly of partiall alkylated dextran-graft-poly[(2-dimethylamino)ethyl methacrylate] copolymer facilitating hydrophobic/hydrophilic drug delivery and improving conetwork hydrogel properties. Biomacromoleculus.

[4] Nutan B., Chandel A.K., Bhanlani D.V., Jewrajka S.K. (2017). Synthesis and tailoring the degradation of multi-responsive amphiphilic conetwork gels and hydrogels fo poly(β-amino ester) and poly(amido amine). Polymer.

[5] Chandel A.K.S., Bera A., Nutan B., Jewrajka S.K. (2016). Reactive compatibilizer mediated precise synthesis and application of stimuli responsive polysaccharides-pollycaprolactone amphiphilic co-network gels. Polymer.

[6] Hosseini M.S., Nabid M.R. (2020). Synthesis of chemically cross-linked hydrogel films based on basil seed (Ocimum basilicum L.) mucilage for wound dressing drug delivery applications. Int. J. Biol. Macromol..

[7] Khampieng T., Wongkittithavorn S., Chaiarwut S., Ekabutr P., Pavasant P., Supaphol P. (2018). Silver nanoparticles-based hydrogel: Characterization of material parameters for pressure ulcer dressing applications. J. Drug Deliv. Sci. Technol..

[8] Choi Y.H., Kim S.-H., Kim I.-S., Kim K., Kwon S.K., Hwang N.S. (2019). Gelatin-based micro-hydrogel carrying genetically engineered human endothelial cells for neovascularization. Acta Biomater..

[9] Javanbakht V., Shafiei R. (2020). Preparation and performance of alginate/basil seed mucilage biocomposite for removal of eriochrome black T dye from aqueous solution. Int. J. Biol. Macromol..

[10] Tosif M.M., Najda A., Bains A., Kaushik R., Dhull S.B., Chawla P., Walasek-Janusz M. (2021). A Comprehensive Review on Plant-Derived Mucilage: Characterization, Functional Properties, Applications, and Its Utilization for Nanocarrier Fabrication. Polymer.

[11] Akbari I., Ghoreishi S.M., Habibi N. (2014). Generation and precipitation of paclitaxel nanoparticles in basil seed mucilage via combination of supercritical gas antisolvent and phase inversion techniques. J. Supercrit. Fluids.

[12] Maqsood H., Uroos M., Muazzam R., Naz S., Muhammad N. (2020). Extraction of basil seed mucilage using ionic liquid and preparation of AuNps/mucilage nanocomposite for catalytic degradation of dye. Int. J. Biol. Macromol..

[13] Wang H., Gong X., Guo X., Liu C., Fan Y.-Y., Zhang J., Niu B., Li W. (2019). Characterization, release, and antioxidant activity of curcumin-loaded sodium alginate/ZnO hydrogel beads. Int. J. Biol. Macromol..

[14] Hua S., Ma H., Li X., Yang H., Wang A. (2010). pH-sensitive sodium alginate/poly(vinyl alcohol) hydrogel beads prepared by combined Ca2+ crosslinking and freeze-thawing cycles for controlled release of diclofenac sodium. Int. J. Biol. Macromol..

[15] Sharifianjazi F., Irani M., Esmaeilkhanian A., Bazli L., Asl M.S., Jang H.W., Kim S.Y., Ramakrishna S., Shokouhimehr M., Varma R.S. (2021). Polymer incorporated magnetic nanoparticles: Applications for magnetoresponsive targeted drug delivery. Mater. Sci. Eng. B.

[16] Aisida S.O., Akpa P.A., Ahmad I., Zhao T., Maaza M., Ezema F.I. (2020). Bio-inspired encapsulation and functionalization of iron oxide nanoparticles for biomedical applications. Eur. Polym. J..

[17] Dacrory S., Moussa M., Turky G., Kamel S. (2020). In situ synthesis of Fe_3_O_4_@ cyanoethyl cellulose composite as antimicrobial and semiconducting film. Carbohydr. Polym..

[18] Supramaniam J., Adnan R., Mohd Kaus N.H., Bushra R. (2018). Magnetic nanocellulose alginate hydrogel beads as potential drug delivery system. Int. J. Biol. Macromol..

[19] Mohammadi R., Saboury A., Javanbakht S., Foroutan R., Shaabani A. (2021). Carboxymethylcellulose/polyacrylic acid/starch-modified Fe_3_O_4_ interpenetrating magnetic nanocomposite hydrogel beads as pH-sensitive carrier for oral anticancer drug delivery system. Eur. Polym. J..

[20] Tavares Luiz M., Santos Rosa Viegas J., Palma Abriata J., Viegas F., Testa Moura de Carvalho Vicentini F., Lopes Badra Bentley M.V., Chorilli M., Maldonado Marchetti J., Tapia-Blácido D.R. (2021). Design of experiments (DoE) to develop and to optimize nanoparticles as drug delivery systems. Eur. J. Pharm. Biopharm..

[21] Janaum N., Butsiri T., Kasemsiri P., Souvanh M., Pongsa U., Theerakulpisut S., Hiziroglu S., Okhawilai M. (2020). Multi Response Optimization of Bioactive Starch Foam Composite Using Taguchi’s Method and Grey Relational Analysis. J. Polym. Environ..

[22] Ounkaew A., Kasemsiri P., Pongsa U., Hiziroglu S., Pasuwan P., Boonlai Y., Theerakulpisut S. (2022). Multiple Response Optimization of Poly(vinyl alcohol)/Starch Based Bioactive Composite Films for Antimicrobial Packaging Applications. J. Polym. Environ..

[23] Shafiee S., Ahangar H.A., Saffar A. (2019). Taguchi method optimization for synthesis of Fe_3_O_4_ @chitosan/Tragacanth Gum nanocomposite as a drug delivery system. Carbohydr. Polym..

[24] Tantiwatcharothai S., Prachayawarakorn J. (2019). Characterization of an antibacterial wound dressing from basil seed (Ocimum basilicum L.) mucilage-ZnO nanocomposite. Int. J. Biol. Macromol..

[25] Glukhova S.A., Molchanov V.S., Chesnokov Y.M., Lokshin B.V., Kharitonova E.P., Philippova O.E. (2022). Green nanocomposite gels based on binary network of sodium alginate and percolating halloysite clay nanotubes for 3D printing. Carbohydr. Polym..

[26] Pooresmaeil M., Javanbakht S., Nia S.B., Namazi H. (2020). Carboxymethyl cellulose/mesoporous magnetic graphene oxide as a safe and sustained ibuprofen delivery bio-system: Synthesis, characterization, and study of drug release kinetic. Colloids Surf. A Physicochem. Eng. Asp..

[27] Amini-Fazl M.S., Mohammadi R., Kheiri K. (2019). 5-Fluorouracil loaded chitosan/polyacrylic acid/Fe3O4 magnetic nanocomposite hydrogel as a potential anticancer drug delivery system. Int. J. Biol. Macromol..

[28] Kurd F., Fathi M., Shekarchizadeh H. (2017). Basil seed mucilage as a new source for electrospinning: Production and physicochemical characterization. Int. J. Biol. Macromol..

[29] Ounkaew A., Janaum N., Kasemsiri P., Okhawilai M., Hiziroglu S., Chindaprasirt P. (2021). Synergistic effect of starch/polyvinyl alcohol/citric acid films decorated with in-situ green-synthesized nano silver on bioactive packaging films. J. Environ. Chem. Eng..

[30] Ray S., Banerjee S., Maiti S., Laha B., Barik S., Sa B., Bhattacharyya U.K. (2010). Novel interpenetrating network microspheres of xanthan gum–poly(vinyl alcohol) for the delivery of diclofenac sodium to the intestine—In vitro and in vivo evaluation. Drug Deliv..

[31] Bi L., Chen Z., Li L., Kang J., Zhao S., Wang B., Yan P., Li Y., Zhang X., Shen J. (2021). Selective adsorption and enhanced photodegradation of diclofenac in water by molecularly imprinted TiO_2_. J. Hazard. Mater..

[32] Pan X., Cheng S., Su T., Zuo G., Zhao W., Qi X., Wei W., Dong W. (2019). Fenton-like catalyst Fe_3_O_4_@polydopamine-MnO_2_ for enhancing removal of methylene blue in wastewater. Colloids Surf. B Biointerfaces.

[33] Javanbakht S., Shadi M., Mohammadian R., Shaabani A., Ghorbani M., Rabiee G., Amini M.M. (2020). Preparation of Fe_3_O_4_@SiO_2_@Tannic acid double core-shell magnetic nanoparticles via the Ugi multicomponent reaction strategy as a pH-responsive co-delivery of doxorubicin and methotrexate. Mater. Chem. Phys..

[34] Yang L., Tian J., Meng J., Zhao R., Li C., Ma J., Jin T. (2018). Modification and Characterization of Fe_3_O_4_ Nanoparticles for Use in Adsorption of Alkaloids. Molecules.

[35] Tantiwatcharothai S., Prachayawarakorn J. (2020). Property improvement of antibacterial wound dressing from basil seed (*O. basilicum* L.) mucilage- ZnO nanocomposite by borax crosslinking. Carbohydr. Polym..

[36] Reddy S.G., Pandit A.S. (2013). Biodegradable sodium alginate and lignosulphonic acid blends: Characterization and swelling studies. Polimeros.

[37] Mahdavinia G.R., Rahmani Z., Karami S., Pourjavadi A. (2014). Magnetic/pH-sensitive κ-carrageenan/sodium alginate hydrogel nanocomposite beads: Preparation, swelling behavior, and drug delivery. J. Biomater. Sci. Polym. Ed..

[38] Chen S.-C., Wu Y.-C., Mi F.-L., Lin Y.-H., Yu L.-C., Sung H.-W. (2004). A novel pH-sensitive hydrogel composed of N,O-carboxymethyl chitosan and alginate cross-linked by genipin for protein drug delivery. J. Control. Release.

[39] Ecke A., Westphalen T., Hornung J., Voetz M., Schneider R.J. (2022). A rapid magnetic bead-based immunoassay for sensitive determination of diclofenac. Anal. Bioanal. Chem..

[40] Cong Z., Shi Y., Wang Y., Wang Y., Niu J., Chen N., Xue H. (2018). A novel controlled drug delivery system based on alginate hydrogel/chitosan micelle composites. Int. J. Biol. Macromol..

[41] Huang X., Xiao Y., Lang M. (2012). Micelles/sodium-alginate composite gel beads: A new matrix for oral drug delivery of indomethacin. Carbohydr. Polym..

[42] Yin Z.-C., Wang Y.-L., Wang K. (2018). A pH-responsive composite hydrogel beads based on agar and alginate for oral drug delivery. J. Drug Deliv. Sci. Technol..

[43] Xie C.-X., Tian T.-C., Yu S.-T., Li L. (2019). pH-sensitive hydrogel based on carboxymethyl chitosan/sodium alginate and its application for drug delivery. J. Appl. Polym. Sci..

[44] Naderi Z., Azizian J., Moniri E., Farhadyar N. (2020). Synthesis and Characterization of Carboxymethyl Cellulose/β-Cyclodextrin/Chitosan Hydrogels and Investigating the Effect of Magnetic Nanoparticles (Fe_3_O_4_) on a Novel Carrier for a Controlled Release of Methotrexate as Drug Delivery. J. Inorg. Organomet. Polym. Mater..

[45] Omer A.M., Ahmed M.S., El-Subruiti G.M., Khalifa R.E., Eltaweil A.S. (2021). pH-Sensitive Alginate/Carboxymethyl Chitosan/Aminated Chitosan Microcapsules for Efficient Encapsulation and Delivery of Diclofenac Sodium. Pharmer.

[46] Guo T., Pei Y., Tang K., He X., Huang J., Wang F. (2017). Mechanical and drug release properties of alginate beads reinforced with cellulose. J. Appl. Polym. Sci..

[47] Tungmunnithum D., Thongboonyou A., Pholboon A., Yangsabai A. (2018). Flavonoids and Other Phenolic Compounds from Medicinal Plants for Pharmaceutical and Medical Aspects: An Overview. Medicines.

[48] Calderón Bravo H., Vera Céspedes N., Zura-Bravo L., Muñoz L.A. (2021). Basil Seeds as a Novel Food, Source of Nutrients and Functional Ingredients with Beneficial Properties: A Review. Foods.

[49] Pandya V.J., Rathod P.P. (2020). Optimization of Mechanical Properties of Green Composites by Gray Relational Analysis. Mater. Today Proc..

[50] Tan L.S., Tan H.L., Deekonda K., Wong Y.Y., Muniyandy S., Hashim K., Pushpamalar J. (2021). Fabrication of radiation cross-linked diclofenac sodium loaded carboxymethyl sago pulp/chitosan hydrogel for enteric and sustained drug delivery. Carbohydr. Polym. Technol. Appl..

[51] Suhail M., Khan A., Rosenholm J.M., Minhas M.U., Wu P.C. (2021). Fabrication and characterization of diclofenac sodium loaded hydrogels of sodium alginate as sustained release carrier. Gels.

[52] Li Y., Wang C., Luan Y., Liu W., Chen T., Liu P., Liu Z. (2022). Preparation of pH-responsive cellulose nanofibril/sodium alginate based hydrogels for drug release. J. Appl. Polym. Sci..

[53] Sadri M., Mohammadi A., Hosseini H. (2016). Drug release rate and kinetic investigation of composite polymeric nanofibers. Nanomed. Res. J..

[54] Nawaz S., Khan S., Farooq U., Haider M.S., Ranjha N.M., Rasul A., Nawaz A., Arshad N., Hameed R. (2018). Biocompatible hydrogels for the controlled delivery of anti-hypertensive agent: Development, characterization and in vitro evaluation. Des. Monomers Polym..

[55] Suhail M., Vu Q.L., Wu P.-C. (2022). Formulation, Characterization, and In Vitro Drug Release Study of β-Cyclodextrin-Based Smart Hydrogels. Gels.

[56] Anwar H., Ahmad M., Minhas M.U., Rehmani S. (2017). Alginate-polyvinyl alcohol based interpenetrating polymer network for prolonged drug therapy, Optimization and in-vitro characterization. Carbohydr. Polym..

[57] Khan M.A., Azad A.K., Safdar M., Nawaz A., Akhlaq M., Paul P., Hossain M.K., Rahman M.H., Baty R.S., El-kott A.F. (2022). Synthesis and Characterization of Acrylamide/Acrylic Acid Co-Polymers and Glutaraldehyde Crosslinked pH-Sensitive Hydrogels. Gels.

[58] Brand-Williams W., Cuvelier M.E., Berset C. (1995). Use of a free radical method to evaluate antioxidant activity. LWT-Food Sci. Technol..

[59] Dharmalingam K., Anandalakshmi R. (2019). Fabrication, characterization and drug loading efficiency of citric acid crosslinked NaCMC-HPMC hydrogel films for wound healing drug delivery applications. Int. J. Biol. Macromol..

[60] Kasemsiri P., Dulsang N., Pongsa U., Hiziroglu S., Chindaprasirt P. (2017). Optimization of Biodegradable Foam Composites from Cassava Starch, Oil Palm Fiber, Chitosan and Palm Oil Using Taguchi Method and Grey Relational Analysis. J. Polym. Environ..

[61] Arifeen W.U., Kim M., Choi J., Yoo K., Kurniawan R., Ko T.J. (2019). Optimization of porosity and tensile strength of electrospun polyacrylonitrile nanofibrous membranes. Mater. Chem. Phys..

