# Supplementary material for: Multi-Responsive Optimization of Novel pH-Sensitive Hydrogel Beads Based on Basil Seed Mucilage, Alginate, and Magnetic Particles
Source: Gels. 2022 Apr 27;8(5):274. doi: 10.3390/gels8050274 (PMC9141934; doi:10.3390/gels8050274)
Supplement: Supplementary file 1 [file gels-08-00274-s001.zip › gels-1658256-supplementary.pdf]

gels

Article

Multi-Responsive Optimization of Novel pH-Sensitive Hydrogel Beads Based on Basil Seed Mucilage, Alginate, and Magnetic Particles

Natwat Srikhao [1], Korrapat Chirochrapas [1], Nessaraporn Kwansanei [1], Pornnapa Kasemsiri [1,*], Artjima Ounkaew [1], Manunya Okhawilai [2,3,*], Chutiwat Likitaporn [4], Somnuk Theerakulpisut [5] and Hiroshi Uyama [6]

Supplementary information

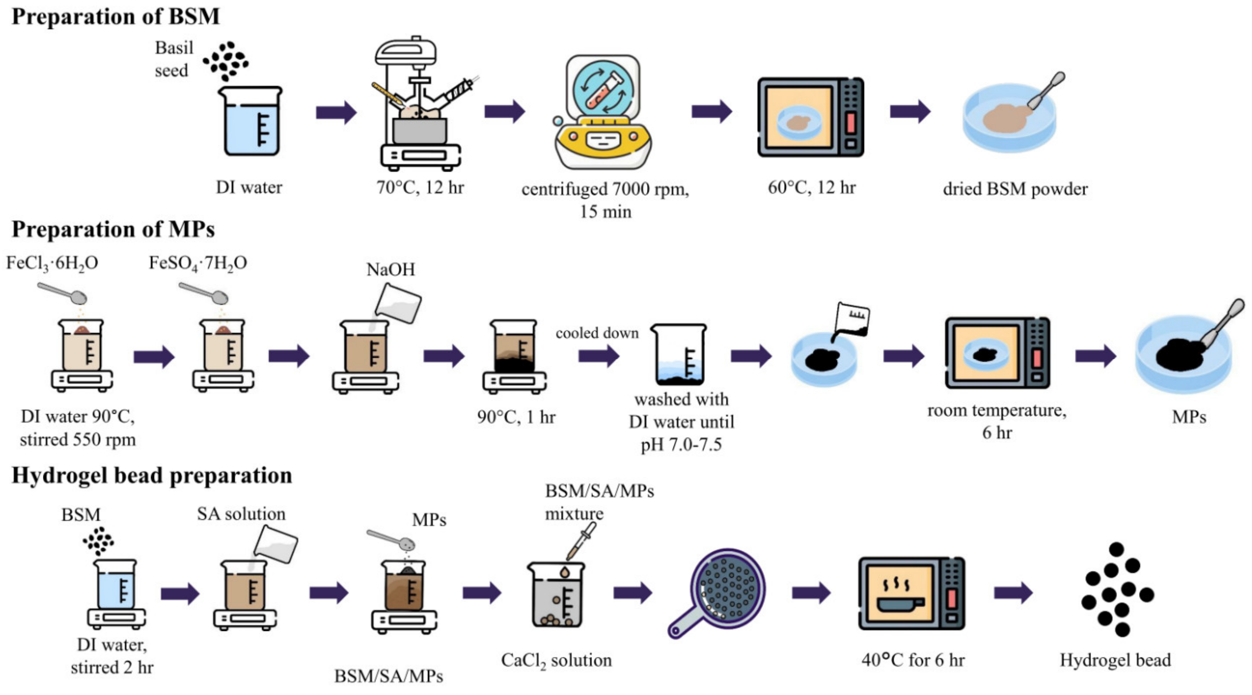

Scheme 1. Overall steps for hydrogel bead preparation.

Table S1. The properties for Grey relational coefficient calculation for the L16 comparability sequences.

Experimental design No.	Gray relational Coefficient						Grey relational grade
	pH 1.2 drug release	pH 7.4 drug release	pH 1.2 antioxidant	pH 7.4 antioxidant	Drug loading	Swelling	
1	0.896	0.353	0.605	0.390	0.334	0.436	0.502
2	0.877	0.339	0.666	0.342	0.334	0.413	0.495
3	1.000	0.402	0.333	0.403	0.334	0.751	0.537
4	0.947	0.366	0.833	0.970	0.334	0.636	0.681
5	0.527	0.333	0.700	0.401	0.334	0.333	0.438
6	0.397	0.354	0.889	0.337	0.334	0.400	0.452
7	0.444	0.601	0.310	0.519	0.334	1.000	0.535
8	0.477	0.369	0.627	0.356	0.334	0.806	0.495
9	0.538	0.343	0.699	0.415	0.334	0.343	0.445
10	0.458	0.366	0.314	0.409	0.334	0.549	0.405
11	0.478	0.476	0.527	0.333	0.333	0.389	0.423
12	0.403	0.358	0.509	0.429	0.334	0.458	0.415
13	0.402	0.363	0.521	0.376	0.334	0.511	0.418
14	0.439	0.472	0.416	0.632	0.334	0.640	0.489
15	0.383	0.354	0.451	0.345	0.334	0.448	0.386
16	0.333	1.000	1.000	1.000	0.333	0.556	0.704

Table S2. Sol fraction of the hydrogel beads.

Experimental design No.	Sol fraction (%)	SD
1	49.55	5.51
2	45.29	2.06
3	40.75	6.66
4	38.28	4.80
5	31.96	5.83
6	36.50	6.05
7	22.23	1.00
8	32.89	1.60
9	17.52	0.11
10	13.33	2.14
11	30.27	1.92
12	30.43	3.67
13	7.05	1.40
14	21.23	2.39
15	15.46	5.83
16	23.26	2.80

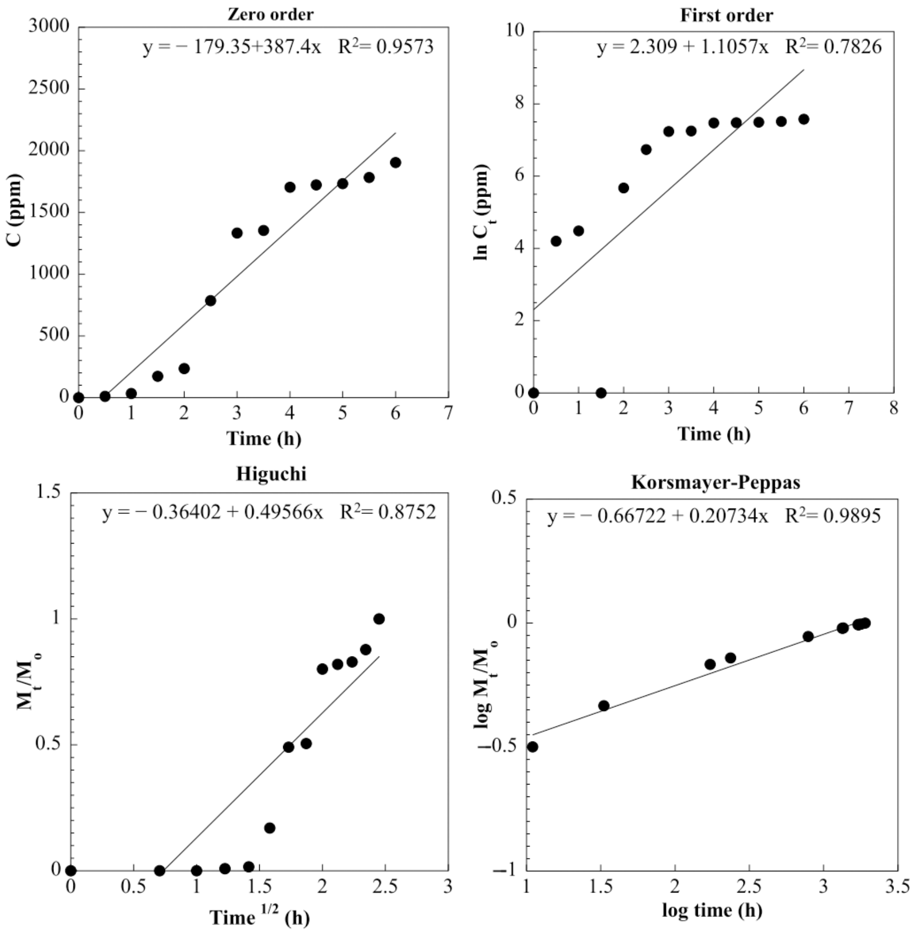

Figure S1. shows the release profiles obtained using the four mathematical models.
